# Establishment of Chronic Infection: *Brucella's* Stealth Strategy

**DOI:** 10.3389/fcimb.2016.00030

**Published:** 2016-03-15

**Authors:** Waqas Ahmed, Ke Zheng, Zheng-Fei Liu

**Affiliations:** State Key Laboratory of Agricultural Microbiology, College of Veterinary Medicine, Huazhong Agricultural UniversityWuhan, China

**Keywords:** *Brucella*, “Stealth” strategy, chronic infection, innate immunity, adaptive immunity, autophagy, apoptosis, small noncoding RNA

## Abstract

*Brucella* is a facultative intracellular pathogen that causes zoonotic infection known as brucellosis which results in abortion and infertility in natural host. Humans, especially in low income countries, can acquire infection by direct contact with infected animal or by consumption of animal products and show high morbidity, severe economic losses and public health problems. However for survival, host cells develop complex immune mechanisms to defeat and battle against attacking pathogens and maintain a balance between host resistance and *Brucella* virulence. On the other hand as a successful intracellular pathogen, *Brucella* has evolved multiple strategies to evade immune response mechanisms to establish persistent infection and replication within host. In this review, we mainly summarize the “Stealth” strategies employed by *Brucella* to modulate innate and the adaptive immune systems, autophagy, apoptosis and possible role of small noncoding RNA in the establishment of chronic infection. The purpose of this review is to give an overview for recent understanding how this pathogen evades immune response mechanisms of host, which will facilitate to understanding the pathogenesis of brucellosis and the development of novel, more effective therapeutic approaches to treat brucellosis.

## Introduction

*Brucella* species are the causative agent of brucellosis, among one of major bacterial zoonotic diseases. *Brucella* is Gram-negative, non-spore-forming, non-motile, facultative anaerobe and intracellular in nature which mainly affect the reproductive tract and cause abortion and infertility in natural host (D'Anastasio et al., 2011). According to host specificity there are 10 species of *Brucella*: *B. melitensis* (natural hosts: goat and sheep), *B. abortus* (natural host: cattle), *B. suis* (natural host: swine), *B. canis* (natural host: dogs), *B. ovis* (natural host: sheep), *B. neotomae* (natural host: desertmice), *B. cetacea* (natural host: cetacean), *B. pinnipedia* (natural host: seal), *B. microti* (natural host: voles), and *B. inopinata* (natural host: unknown; Whatmore et al., 2007). However, among recognized *Brucella* species the most pathogenic for human include *B. melitensis, B. abortus, B. suis*, and *B. canis*. In addition, *Brucella* represent public health problems in low income countries (Atluri et al., 2011; Martirosyan et al., 2011). Humans can acquire infection by different ways such as direct contact with diseased animals or by consumption of *Brucella* affected animal's products. *Brucella* infection in human is considered as a febrile illness that can progress into a long lasting disease with the appearance of severe complications (de Figueiredo et al., 2015).

In human and animal brucellosis, persistence occurs in the tissues of mononuclear phagocyte system including bone marrow, lymph nodes, liver and spleen. Additionally, in both human and animal host, *Brucella* may be encountered within bones and joints, as well as in male reproductive organs while in placenta and fetus of females (Atluri et al., 2011; Martirosyan et al., 2011). If brucellosis is not properly treated, it develops into chronic infection that leads to severe health problems resulting in remarkable morbidity and economic loss in endemic areas (in the Middle East, North, Central and South America, north Africa, Mediterranean countries, and countries of the Caucasus and central Asia; Godfroid et al., 2011). Brucellosis is one of the frequently encountered zoonotic diseases that infect approximately 500,000 new cases annually (Durward et al., 2012). Even though there are some commercial vaccines are used to control animal brucellosis, yet no safe and effective vaccines are available for humans or pregnant animals. Generally, occurrence of human brucellosis is directly linked with natural hosts, and not transmitted from person to person with exception of two reported cases (Pappas et al., 2006).

Due to certain complications, such as economic issues, ethical aspects and practical problems, it is difficult to study brucellosis in natural hosts, as a consequence mice model is widely used to study the relationship of immune response mechanism with brucellosis. The course of murine brucellosis depends upon bacteria (strain, virulence, dose and inoculation route) and certain host parameters including breed, genetic background, age, sex, and physiological status (Grillo et al., 2012). *Brucella* infection is divided in three steps: in the first step pathogen invades host within 2 days of infection, in second step pathogen replicates within different organs of the reticulo-endothelial and reproductive systems from 2 days to 3 weeks which is known as the acute phase of infection, while in the 3rd step, the pathogen displays differences in the pathology of various tissues and lasts from up to 6 months to 1 year or more, known as chronic phase (Martirosyan et al., 2011; Grillo et al., 2012). During chronic phase, the number of bacteria reaches a maximum level in spleen and liver (from 7 to 12 weeks), followed by the declining chronic phase during which number of bacteria decreases and *Brucella*e are eliminated from spleen and liver (Martirosyan et al., 2011; Martirosyan and Gorvel, 2013). In this review, we mainly summarize the strategies and mechanisms employed by *Brucella* to evade the immune response of host as well as implication of these modulations in the pathogenesis of brucellosis.

## *Brucella* pathogenesis

After internalization, *Brucella* are challenged with harsh diverse environment situations. The pathogen develops different strategies including evasion and resisting intracellular host defense mechanisms for its adaptation that is predicted by certain structural components or presence of virulence factors. The pathogenesis of brucellosis mainly depends upon macrophages, dendritic cells and placental trophoblasts for its survival and replication (Copin et al., 2012).

**Figure 1:**
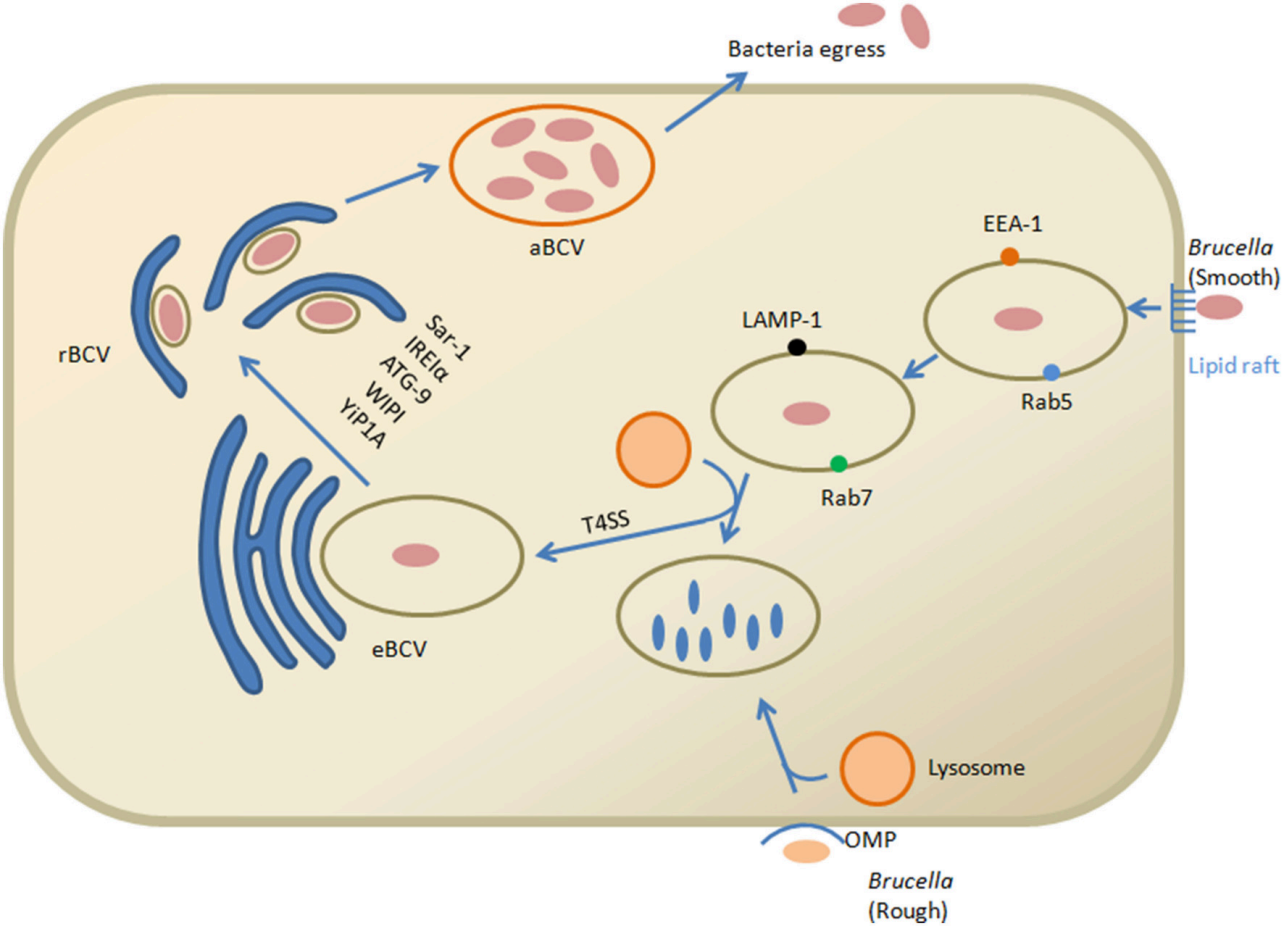
**Model of *Brucella* invasion and intracellular trafficking within macrophage cells**. Smooth *Brucella* and lipid rafts interact on the plasma membrane. *Brucella* derived β-1,2-glucans help in the conversion of external vacuole lipid rich domains that leads to fusion with lysosomes for replication of bacteria. This interaction ultimately activates the T4SS proteins. T4SS proteins in the cytosol of host cell facilitate the interaction with the endoplasmic reticulum that converted into the replicative vacuole. Conversion of the eBCV into rBCV is facilitated by Yip1A dependent activation of IRE1α and results in formation of large vacuoles that depend upon ATG9 and WIPI. The aBCVs formation is dependent on the autophagy initiation proteins, ULK1, ATG14L and BECLIN1, that complete the intracellular cycle and finally through lytic and non-lytic mechanisms the pathogen is released from the cell. While the role of the rough *Brucella* outer membrane protein has been verified, their internalization and intracellular trafficking is unclear but involved in lysosomal degradation.

There are many intracellular host defense mechanisms, among one of them is degradation within the lysosomal compartments. *Brucella* control the intracellular trafficking of their vacuoles, named as the *Brucella*-containing vacuole (BCV) to avoid this degradation (Celli et al., 2003). Fusions of BCVs with membrane elements like endoplasmic reticulum (ER) ultimately favor a safe replicative niche. Internalization of smooth *Brucella* is facilitated in the macrophage cells by lipid raft attachment to the plasma membrane. EEA-1 and Rab5 are the markers of early endocytic pathway associated with the lipid raft containing vacuole. For the maturation of vacuole, these external lipid rafts high in cholesterol are converted into *Brucella*e derived β-1,2-glucans b (von Bargen et al., 2012). Maturation proceeds with the passage of time and early markers present on the BCV are displaced to Rab7 and LAMP-1 subsequently with the interaction to the late endosomal compartments (Figure 1). BCV interact with late endosomes/lysosomes that control vacuolar acidification and transcription of various *Brucella* factors (for example: *vir*B) but result in inhibition of vacuolar attachment of the proteolytic enzyme, cathepsin D (Boschiroli et al., 2002). The type IV secretion system (T4SS) of *Brucella* that encodes virB operon thought to be involved in secretion of several putative bacterial factors and support maturation of the BCV ultimately controls the intracellular and stealthy lifestyle of the pathogen (Roux et al., 2007; Dohmer et al., 2014). Furthermore, other key players that are involved in the intracellular life of *Brucella* include the two-component regulatory system BvrS/BvrR, the cyclic b-glucan, the LuxR-like transcriptional regulator VjbR and the *Brucella* lipopolysaccharide (LPS) (Sola-Landa et al., 1998; Arellano-Reynoso et al., 2005; Weeks et al., 2010). Recent studies have identified some new players such as the flagellum-like structure, the transporter-like protein BacA and phosphatidylcholine required for intracellular survival within host cells (Roop et al., 2009). In addition to *Brucella* T4SS, the CD98hc transmembrane protein is recognized as essential for intracellular proliferation and modulates different signaling pathways (Keriel et al., 2015).

Calreticulin is an antigen of endoplasm reticulum that also favors replication in strict rBCVs (replicative *Brucella*-containing vacuole). Autophagy proteins play an important role in the production of (autophagic *Brucella*-containing vacuole aBCVs) and pathogen observed in aBCVs contain LAMP1 at the later stage after infection (48–72 h). Finally through the lytic and non-lytic mechanisms, the pathogen is released from the cell (Boschiroli et al., 2002). However, when *Brucella* reach the ER not only provides a safe niche but it also protects the pathogen from strong bactericidal action of phagocytic cells. New insight in biology of pathogenesis of brucellosis shed lights that *Brucella* utilizes cell-cycle control system for survival within host intracellular environment similar to *Caulobacter crescentus. Brucella* is blocked at the G1 stage of growth and resumed replication starts again after reaching within the intracellular compartments (De Bolle et al., 2015). Correspondingly, *Brucella* is well equipped to tolerate both physiologic and metabolic stresses that essential for its virulence (Kohler et al., 2003).

## *Brucella* modulation of innate immune response

As an effective pathogen, *Brucella* has developed well organized strategies that allow it to evolve and interfere with innate immune recognition which ultimately favor the environment for generating adaptive immune response (Diacovich and Gorvel, 2010). The first line of defense against brucellosis include phagocytosis by cells (neutrophils, macrophages and dendritic cells (DC), and natural killer (NK)-cells), different secretion like cytokines and chemokines, recognition of molecules typical for microbe's pathogen-associated molecular patterns (PAMPs) by pattern-recognition receptors (PRRs), and activation of the complement system (Diacovich and Gorvel, 2010).

Neutrophils are one of the most significant short-lived phagocytic cells in innate immune response against microbial pathogens. However, in brucellosis these cells are not stimulated for effective degranulation. Even though, this pathogen does not replicate within these cells, it can survive at initial stage of infection (Riley and Robertson, 1984) and resists killing (Barquero-Calvo et al., 2007). *Brucella* have different defensive antimicrobial resistance mechanisms against hypohalide, phospholipase A2, cathelicidin, lysozyme, and defensins like reactive oxygen intermediates (ROI) and reactive nitrogen intermediates (RNI) which ultimately help this pathogen during transportation to lymphoid tissues facilitated by neutrophils (Nathan and Shiloh, 2000).

The host ability to identify *Brucella* as Gram negative bacteria via TLR2, TLR4, and TLR5 are reduced (Iwasaki and Medzhitov, 2004). Consequently, *Brucella* avoid the generation of specific host response by displaying wrong “bar code” such as neutrophil infiltration at the infection site. *Brucella* have the ability to survive *in vivo* that is not increased by NADPH oxidase and neutrophils (even though it can evade them; Barquero-Calvo et al., 2015).

Other than neutrophils, activated NK cells also kill infected targets and act as first line of defense against *Brucella* (Fernandes et al., 1995). *Brucella* activate NK cells by inducing antigen presenting cells to release IL-2 and NK cells are converted into killer cells by IL-2 activation, secretion of IFN-γ and production of IFN-γ which plays an important role in developing a Th1- or Tc1-like response (Gao et al., 2011).

Macrophages and dendritic cells are considered to be key elements of the innate immune response against the intracellular bacteria like *Brucella*. Within the first few hours after entry, 80-90% pathogens are killed by macrophages and DCs, while some of them reach the replicative niche as surviving-pathogens (Watarai et al., 2002). *Brucella* use lipid rafts to enter within both murine macrophages and human monocytes while it uses PI3-kinase and TLR4 receptors to enter DCs (Pei et al., 2008).

The innate immune system recognizes pathogens in various tissues facilitated by PRRs which includes toll-like receptors (TLRs; Iwasaki and Medzhitov, 2004), Nod-like receptors (NLRs; Franchi et al., 2008), RIG-like receptors (RLRs), and complement (Snyderman et al., 1968). By the detection of these receptors, host cells differentiate bacteria from virus by recognizing PAMPs (Hoebe et al., 2004). There are some distinctive products in bacteria such as lipopolysaccharides, flagellin, lipoteichoic acids, and lipoproteins that are recognized by TLR2, TLR4, TLR6, TLR1, and TLR5 receptors (Iwasaki and Medzhitov, 2004). Interaction with bacterial surface carbohydrates (LPS of Gram-negative bacteria) helps in activation of alternative complement pathway.

*Brucella* produces lipid A that has significant functions in immune evasion mechanism via TLR4. In comparison with other enterobacterial LPS (C_12_–C_16_), the pathogen contains longer fatty acid residue (C_28_) and due to this modification in LPS structure which reduces its endotoxic properties by decreasing TLR4 agonist action (Lapaque et al., 2009). In *B. abortus* mutants, failure in the addition of lipid A with C_28_ acyl chain consequently leads to severe inflammatory condition than wild-type parent strain and contribute to decreased infectivity within BALB/c mice and in macrophages as well (Parent et al., 2007). In addition to evasion of TLR4, *Brucella* produce flagellin which plays a role in evasion sensing by TLR5 that has no specific TLR5 agonist domain (Andersen-Nissen et al., 2005).

**Figure 2:**
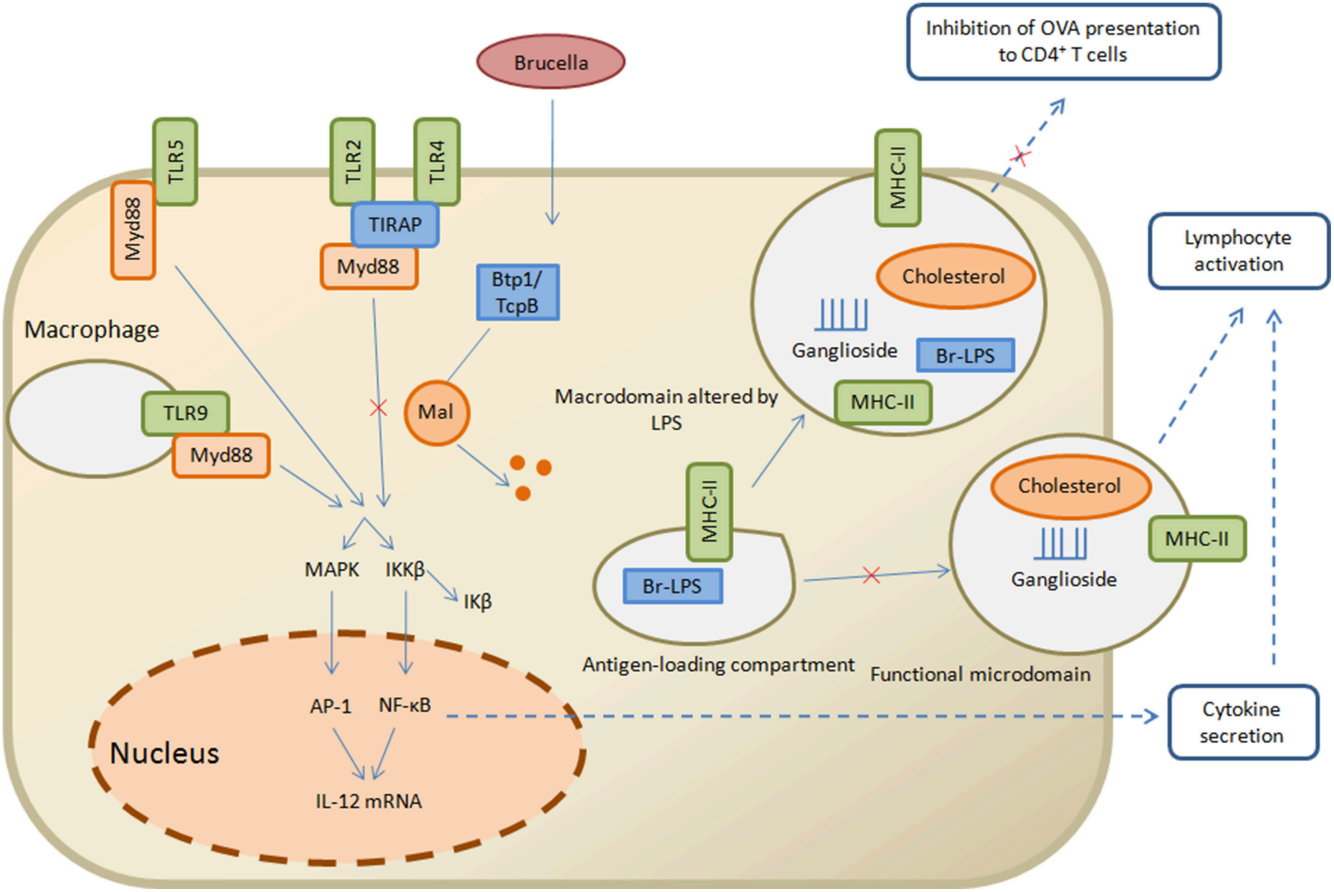
**Strategies of *Brucella* to evade the innate and adaptive immunity**. *Brucella* Btp1/TcpB is proposed to act in host cytosol where it interferes with TLR signaling pathway and facilitates ubiquitination and degradation of Mal which in turn inhibits TLR2 and TLR4 pathway, resulting in inhibition of NF-κB secretion and lymphocyte activation. On the other hand, the antigen loading compartment, comprised of Br-LPS and MHC class II molecules, is capable of interacting with functional microdomain which result in lymphocyte activation and inhibition of OVA presentation to CD4+ T cells. Straight arrows indicate the innate immunity pathways while dashed arrows are related to adaptive immunity pathways.

*Brucella* use other evasion strategies by suppressing innate immune signaling other than producing PAMPs with decreased TLR agonist activity via TIR domain-containing protein designated as Btp1 in *B. abortus* and TcpB in *B. melitensis*. However, the detailed mechanism of this protein is still incompletely understood, but evidence suggests that for binding with TIR domain-containing adaptor protein (TIRAP), it competes with myeloid differentiation response gene 88 (MyD88) that ultimately facilitates ubiquitination and degradation of Mal and inhibits both TLR4 and TLR2 signaling (Figure 2; Snyder et al., 2014). Maturation of the dendritic cells and production of pro-inflammatory cytokines like IL-12 and TNF-α after *Brucella* invasion and internalization is reduced due to this TLR-inhibition (Salcedo et al., 2008). During the early stage of infection Btp1/TcpB seems to be important in the immune-evasive activity, such that at the inoculation site in immune deficient IRF1 mice, there is no systemic spread by *B. melitensis tcpB* mutant. The weakened phenotype of *tcpB* mutants for immune-competent mice suggests that *Brucella* have ability to evade innate immunity by multiple strategies (Radhakrishnan et al., 2009).

As a stealth invader, *Brucella* LPS also play a pivotal role in reducing deposition of complement constituent C3 (Barquero-Calvo et al., 2007). C3 covalently binds with hydroxyl residues present on the surface of bacteria. Many pathogens like *Salmonella Typhimurium* have free hydroxyl residues in their O-polysaccharide that favors binding with C3 while *B. abortus* has linear homopolymer of 1,2-linked 4,6-dideoxy-4-formamido-alpha-d-mannopyranosyl residues in its O-polysaccharide (Caroff et al., 1984). Generation of pro-inflammatory complement products like C3a and C5a is prevented by C3 binding to *Brucella* O-antigen. Different studies have shown O-antigen plays an important role in facilitating a non-inflammatory response through lipid raft microdomains and macrophage class A scavenger receptor (SR-A; Kim et al., 2004). These studies suggest that key strategies used by *Brucella* to modulate innate immune mechanism are inhibition to TLR, complement system and involvement of phagocytic cells. However, further studies on maturation of DCs *in vivo* and the contribution of newly identified protein in the innate immune response as well as their effect on adaptive immune response will be required to more fully understand the establishment of chronic infection.

## *Brucella* impressive mechanisms to evade adaptive immune response

Even though innate immunity efficiently controls the replication of *Brucella* at acute phase of infection, a well-organized adaptive immune response is also essential for the chronic stage (Baldwin and Goenka, 2006). Furthermore, *Brucella* has developed multiple strategies to defeat host defense mechanisms and consequently confirm the establishment of chronic infection (Monack et al., 2004).

For the initiation and control of adaptive immune responses, some cells such as DCs play a significant role (Kapsenberg, 2003). On the other hand, intracellular pathogens have established multiple mechanisms to challenge the function of DCs that ultimately facilitates the pathogen's entry into the host. *Brucella* has established several strategies which confirm its shifting from innate immune system to the adaptive immune system for evasion into the host immune machinery. Some recent evidence have confirmed the efficient proliferation of this pathogen within DCs both *in vitro* (Archambaud et al., 2010) and *in vivo* (Salcedo et al., 2008). Besides the proliferation, hindrance of maturation of DCs by *B. abortus* 2308 and *B. suis* 1330 also reported by *in vitro* studies (Billard et al., 2007; Salcedo et al., 2008). In addition, prior studies have shown that expression of MHC class II, CD80 and CD86 were decreased in the *Brucella* infected DCs. Incompetent antigen presentation to naïve T cells and inhibition of maturation result in inhibiting secretion of pro-inflammatory cytokines such as IL-12 band TNF-α (Billard et al., 2007). In long lasting vaccine induced immunity, memory adaptive immune responses are important. For clearance of *Brucella*, IFN-γ-mediated type I immune responses are essential (Goenka et al., 2011). *Brucella* modulates MHC-I and MHC-II expression prompted by IFN-γ, a mechanism that depends on cytokine regulation (Gentilini et al., 2015), which results in inhibition of MHC-I and MHC-II molecules (Barrionuevo et al., 2008, 2013).

In brucellosis, adaptive immune response mechanisms are divided in three main steps: in the first step it inhibit the intracellular survival of *Brucella*, IFN-γ produced by CD4+, CD8+, and T cells which initiates the bactericidal function in macrophages. Secondly, infected macrophages are killed which lead to cytotoxicity by CD8+, and T cells. Thirdly, in the endocytic compartments opsonization of *Brucella* occur by IgG2a and IgG3 to enhance phagocytosis (Goenka et al., 2011; Martirosyan et al., 2011). Additionally, IL-12, IFN-γ, and TNF-α are key cytokines in brucellosis, initiating innate and adaptive immune response and giving directions to immune-associated cells (Martirosyan et al., 2011; Durward et al., 2012). Furthermore, *Brucella* LPS also successfully enhances Th1-type cytokine response such as IL-10 and IFN-γ (Kianmehr et al., 2015) while 5-Lipoxygenase down regulates the manifestation of Th-1 immune response particularly IL-12 in *Brucella* infection in macrophages (Fahel et al., 2015).

*Brucella* has a unique property to survive within the host which results in the establishment of chronic infection. Therefore, strong immune regulation events are essentially required in brucellosis. In chronic brucellosis, immunosuppressive state establishes with the concomitant increase of CD4+, CD25+ T cells in spleen, which ultimately plays a significant role in the regulation of effector T lymphocytes (Pasquali et al., 2010). These murine CD4+ or CD25+ T cells encourage the elimination of *Brucella* from infected by antibody depletion (Pasquali et al., 2010). In comparison MHC class II and CD4+ Ab-deficient mutant mice are more able to eliminate *Brucella* than in wild type mice. The decrease of Møs and DCs recruitment causes reduced activation of CD8+ T lymphocytes that results in immunosuppression (Hort et al., 2003) and deficient clearance of *Brucella* from spleens, lymph nodes and livers (Rolan and Tsolis, 2007).

In addition to this, *Brucella* interfere with the establishment of protective Th1 immune response by avoiding secretion of IL-12 and preventing the T-cell stimulatory action of infected DCs (Salcedo et al., 2008). As a result, maturation capacity of DCs is reduced and characterized by decreased expression of MHC class II indicating co-stimulatory molecules on the external cell surface are necessary to present exogenous protein antigens to particular T cells (Billard et al., 2007; Salcedo et al., 2008). Mature DCs are capable of having unique tolerogenic properties, and *Brucella* inhibits complete activation DCs by using this property to subvert immune responses. There is certain evidence that suggests that initiation of tolerance is not limited to undeveloped DCs, however fully mature DCs are able to induce tolerance which ultimately results in the establishment of long-term chronic infection (Lutz and Schuler, 2002). Moreover, contact of immature DCs with CD4+ naïve T cells may induce regulatory T-cell action of Tregs and prevents Th1 response in a TGF-β-dependent manner, this incidence was previously recorded during human chronic brucellosis (Elfaki and Al-Hokail, 2009). Additionally, *Brucella* infected macrophages activate DCs to present antigen which stimulates immune response of host (Billard et al., 2007). Ineffective contact of CD4+ T cells with immature DCs results in failure in delivery of licensing signals required for CD8+ cytotoxic T cell stimulation. Reduction in the level of IL-10 indicates an improvement in host resistance mechanism to brucellosis (Smith et al., 2004). These findings suggest that *Brucella* hinder antigen presentation, and have significant role in the interplay between innate and adaptive immune mechanisms.

*Brucella* LPS has noncononical structural differences that play important roles in its stealthy behavior which in turn helps in the initiation of the adaptive immune response and antigen presentation to T cells (Conde-Alvarez et al., 2012). LPS forms macrodomain clusters after recycling from compartments that are specialized sites for antigen loading and processing to macrophage cell surface. These macrodomain clusters are composed of *Brucella* LPS, MHC class II molecules and lipid rafts. MHC class II molecules form complexes that facilitate the initiation of immune response (Lapaque et al., 2006). Indeed, purified *Brucella* LPS is capable of inhibiting peritoneal macrophages presentation of ovalbumin and hen egg lysozyme antigenic peptides to specific T-cell hybridomas in the presence of MHC class II (Forestier et al., 2000). At the plasma membrane of peritoneal macrophages, Br-LPS moieties sequester with MHC class II molecules where their biogenesis occurs in lysosomes. Similarly, in B lymphocytes, these Br-LPS moieties also aggregate in the MHC class II compartments (Figure 2; Forestier et al., 1999). B-cell proliferation is initiated by the secretion of *Brucella* virulence factor prpA that interact with macrophages and release several soluble factors necessary for establishing chronic infection (Spera et al., 2006, 2013). In addition to this, *Brucella* change the cytokine level of IFN-γ, TNF-α, IL-10 and TGFβ1 in the early stages of brucellosis in a *prpA* dependent manner (Spera et al., 2014). Recent studies clarify the roles of Btp1/TcpB, Br-LPS and PrpA as being significant immunomodulatory molecules with the ability to interplay with host immune mechanisms. Furthermore, they have capability to inhibit the secretion of IFN-γ and increase the secretion of IL-10 that affects Th1 immune response (Wang et al., 2012).

*Brucella* effector molecules are capable of controlling TLR signaling pathway that involved in DC maturation with significant effects on T-lymphocyte activation and antigen presentation. *Brucella* TIR protein 1 (Btp1) shows sequence resemblances with Toll/IL-1 recptors (TIR) domain family. Different studies investigated the role of Btp1 in DC maturation due to significance of TIR domain in the TLR signaling (Kenny and O'Neill, 2008; Atluri et al., 2011). Btp1 inhibits both the production of proinflammatory cytokines and DC maturation that leads to inhibition of TLR2 and TLR4 signaling (Salcedo et al., 2008; Atluri et al., 2011). *Brucella* lumazine synthase also induces negative effect by blocking TLR4-MD2 complex (Rossi et al., 2015). Btp1/TcpB bind adapter TIRAP at the cell membrane and block NF-κB activation (Radhakrishnan and Splitter, 2010). Therefore, *Brucella* inhibits TLR-signaling pathway to reduce the function of infected DCs. Recently, it has been demonstrated that Btp1 associates with host microtubules to protect them from depolymerization an additional function of this protein (Radhakrishnan et al., 2011). Additionally, it has been shown that this protein causes inhibition of CD8+ T-cell killing of *Brucella* target cells and represents an adaptive immune evasion strategy (Durward et al., 2012). Thus, these findings of new effector proteins that are capable of interaction with TLR signaling pathway reinforce the notion that *Brucella* use multiple strategies to evade host adaptive immune system to establish chronic infection (Gorvel, 2008).

## Selective subversion of autophagy pathway

Autophagy, “self-eating,” contributes to several cellular and organism homeostatic mechanisms by capture of cytosolic components, damaged organelles such as mitochondria, protein aggregates (Kraft and Martens, 2012), and intracellular bacteria (whether cytosolic or vacuolar) into specialized double-membrane vacuoles called autophagosomes (Yang and Klionsky, 2010). Even though, autophagy is initially found in nutrient reprocessing in response to starvation, some other cellular and oxidative stress functions are well recognized in the induction of autophagy pathway and also contribute to innate immune response (Levine et al., 2011) through antibacterial activities.

Intracellular pathogens adopt different strategies for involvement in the host autophagic pathway that include damage to the membrane, hiding to avoid recognition by the autophagy mechanism and development of replicative niches (Bestebroer et al., 2013).

For the development of *Brucella* replication (rBCV) fusion of endoplasmic reticulum with endocytic compartment occurs in the Sar1- and Rab2- dependent manner. In macrophages and epithelioid cells during early stages of brucellosis, rBCV convert into a specialized property compartment known as autophagic BCV (aBCV). Recent evidence supports the theory that autophagy-associated proteins play a key role in the biogenesis of rBCV and contribute to completion of intracellular life cycle, *Brucella* subverts the host cell membrane trafficking pathways (Starr et al., 2008, 2012).

However, rBCV to aBCV conversion depends upon autophagy initiation proteins for example BECLIN1, PI3K, ULK1, and Atg14L but independently of autophagy elongation proteins such as Atg5, Atg7, LC3B, Atg4B, and Atg16L1 that are necessary for abolishing the autophagosome formation (Starr et al., 2012). The mechanism involved is still not clearly explained, other studies suggest that ULK1 and Beclin 1 are required for non-canonical pathways but independent of LC3 and Atg7, as well as Atg5 recruitment (Collins et al., 2009; Nishida et al., 2009). Furthermore, aBCV formation depends upon the small GTPase Rab9 which is not required in HeLa cells (Starr et al., 2012). Autophagosome maturation initiates into endocytic compartment when BECLIN1 and PI3K form a complex, but depletion in ATG14L leads to decreased aBCV formation which starts from the endoplasmic reticulum-localized BECLIN1 complex. Recent studies support the non-canonical autophagy pathway playing a key role in host-pathogen interaction, and have common upstream regulators with canonical autophagy pathway (Starr et al., 2012). The egress mechanism is helpful in pathogen release and cell-to-cell spread in which Atg proteins play a significant role in this process (Duran et al., 2010; Manjithaya and Subramani, 2011).

There are some new insights about the role of unfolded protein response (UPR) and autophagy in *Brucella* replication and rBCV biogenesis (de Jong et al., 2013; Celli and Tsolis, 2015), such as activation of IRE1α in macrophages and HeLa cells by *B. abortus* (Taguchi et al., 2015), while ATF6, PERK and IRE1α in the instance of *B. melitensis* infection (Smith et al., 2013). The UPR pathways activation by *Brucella* also supports the idea that IRE1α is important in the bacterial replication (Qin et al., 2008). Yip1A is a host protein that leads to phosphorylation of IRE1α in brucellosis and binding with COPII for localization into Endoplasmic reticulum exist sites. Furthermore, Yip1A and IRE1α are essential for *Brucella* replication and rBCV biogenesis (Taguchi et al., 2015). There is up regulation of Sar1 and COPII by Yip1A-dependent activation of IRE1α, results in formation of large vacuoles that depend upon ATG9 and WIPI (Taguchi et al., 2015). These findings clarify the role of IRE1α in brucellosis and facilitate the formation of autophagic origin vacuoles that convert endosomal *brucella*-containing vacuole (eBCV) into rBCV (Figure 1; Wang et al., 2014).

It is also observed that UPR activation in the *Brucella*-infected macrophages via TcpB facilitates protein folding that affects *Brucella* intracellular growth (Smith et al., 2013). In addition to this some effectors, such as BspC, BspK, BspH, and BspG, can also enhance the endoplasm reticulum stress by ecoptic expression in HeLa cells (Myeni et al., 2013). These results show that the initiation of autophagy proteins is important in conversion of rBCV to aBCV while Yip1A is also required in *Brucella* replication and rBCV biogenesis. Hence, this is an essential strategy used by *Brucella* to combat with host immune response for which further study is required to investigate the involvement of autophagy in *Brucella* pathogenesis as well as in innate and adaptive immune mechanisms.

## Inhibition of apoptosis

Apoptosis is an extremely well-regulated manner of programmed cell death, which is generally facilitated by the initiation of caspases, and excellent host defense response against intracellular bacteria. However, inhibition of apoptosis is another strategy of *Brucella* to maintain intracellular niche for its replication. *Brucella* inhibits apoptotic mechanism in infected macrophages with the induction of chemical stimuli (Gross et al., 2000). Furthermore, in *B. melitensis* infection there is down regulation of genes involved in apoptotic pathway in mitochondria (He et al., 2006). There is significant increase in Nedd4 activity during brucellosis in a specific calcium-dependent manner. *Brucella*-infected macrophages treated with Nedd4 decrease this activity that in turn stops calpain 2 degradation and leads to macrophages apoptosis (Cui et al., 2014). Caspase-2 is involved in the regulation of many genes and pathways which prompts macrophage death that is an important feature of apoptosis and pyroptosis (Bronner et al., 2013). Moreover, upregulation of A20 leads to inhibition of NF-κB that limiting caspase-8-dependent macrophage cell death and favors intracellular growth of bacteria (Wei et al., 2015).

Another important strategy that is employed involves the interaction of *Brucella*-macrophage to enhance virulence by inhibiting macrophage cell death. After the invasion of smooth *Brucella* into macrophages for replication, the pathogen automatically converts into rough mutants that can results in macrophage cytotoxicity that favors bacterial egress and dissemination (Pei et al., 2014). *B. abortus* 2308 rough mutants that lack of surface LPS exhibited failure in the inhibition of apoptosis (Pei et al., 2006), because this depends upon T4SS and is associated with the effect of LPS inhibiting TLR signaling (Pei et al., 2008).

**Figure 3:**
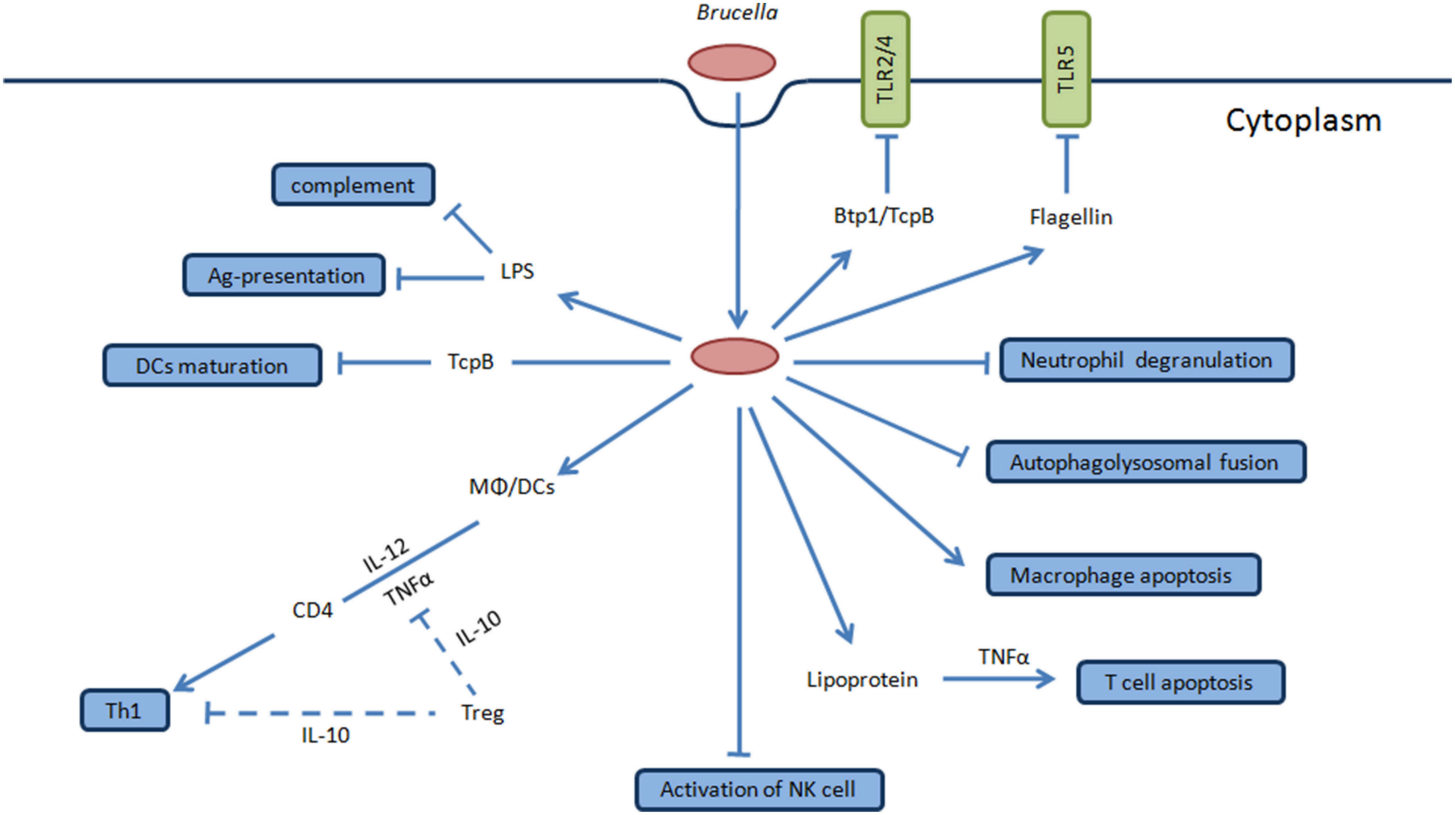
**Overview of stealth strategies of *Brucella***. TLR signaling pathway is involved in the recognition of *Brucella* which mediates the secretion of TNF-α and IL-12 by DCs and Møs during the initial stages of infection that favor intracellular survival and replication. *Brucella* produces Btp1/TcpB protein that inhibits TLR2/4 signaling pathway while flagellin inhibits TLR5. *Brucella* LPS O-antigen binds with C3 preventing activation of complement cascade. *Brucella* inhibits activation of NK cells and neutrophil degranulation to interfere with innate immune response of host. Btp1/TcpB produced by this bacteria inhibit dendritic cells maturation, and *Brucella* LPS inhibits of antigen presentation, coupled together ultimately interfere with innate and adaptive immunity of host facilitating chronic infection. *Brucella* lipoprotein increases apoptosis of T cells which is dependent upon TNF-α secretion that directly inhibits the T cell response. Furthermore, *Brucella* inhibits macrophage apoptosis and autophagolysosomal fusion which are among the key stealth strategies of this pathogen. The final consequence of interfering with these mechanism results in the clinical manifestations of human brucellosis and in the natural hosts.

*Brucella* lipoprotein increases T cells apoptosis which depends upon TNF-α secretion, sheding light on mechanisms that *Brucella* uses to directly inhibit T cells responses, involved in adaptive immune evasion (Figure 3; Velasquez et al., 2012). Additionally, *Brucella*- infected neutrophils and monocytes have obvious upregulation of various adhesion molecules such as CD106 and CD54 that result in inhibition of apoptosis in brucellosis (Scian et al., 2013). Altogether this evidence indicates that apoptosis inhibition is a strategy of *Brucella* for intracellular replication that in turn facilitates evasion of the immune response.

## Putative role of small noncoding RNA against *Brucella* stealth strategy

MicroRNAs are part of small noncoding RNA involved in regulation of gene expression and in cellular mechanisms, apoptosis and signal transduction (He and Hannon, 2004; Djuranovic et al., 2012; Leung and Sharp, 2013). Even though, we are still at the initial stages of knowing the role of miRNAs and how they develop and regulate the immune mechanisms, recent evidence found that miRNAs play a pivotal role in immunity such as miR-181a and miR-223 in establishing and maintaining immune cells (Chen et al., 2004; Matsushima et al., 2011), miR-146 regulate TLR signaling and cytokine response in innate immunity (Tili et al., 2007). Furthermore, they are involved in adaptive immunity associated antigen presentation such as miR-155 (Schulte et al., 2013), and miR-181a in T cell receptor signaling (Iliopoulos et al., 2010).

*Brucella* adapt diverse environmental conditions and use multiple strategies to evade host cell defense. It would seem that *Brucella* sRNA may play a significant role in bacterial responses to stress. Hfq protein mediates most of the sRNA-mRNA interactions which is required for virulence and to control bacterial stress response in pathogen–host interactions (Papenfort and Vogel, 2010; Hanna et al., 2013) as well in *Brucella* stress response (Robertson and Roop, 1999). A recent finding also supports the involvement of hfq in pathogenicity associated invasion and proliferation within host cell as illustrated by a *B.melitensis* hfq mutant (Cui et al., 2013).

Casewell et al. reported that *B. abortus* sRNAs, abcR1 and abcR2 play essential roles in pathogenicity and in chronic infection, resulting in a significant decrease in intracellular survival in a mouse model and in macrophages as well (Caswell et al., 2012). Transcriptional regulators like gntR code for miRNAs required for *Brucella* virulence. However, their inhibited expression within macrophages led to decreased *Brucella* intracellular survival indicating that miRNAs are essential for the adaptation to stress conditions and ultimately cause modulation of *Brucella* intracellular survival (Wang et al., 2015).

Inhibited expression of syntaxin mRNA with small interfering RNAs modulated initial phagocytosis and intracellular survival of *Brucella* (Castaneda-Ramirez et al., 2015). We studied the expression of miRNAs in *B. melitensis*-infected cells and found several miRNAs such as miR-92a, miR-93, let-7b, miR-1981, and miR-181b differentially expressed compared to mock-infected cells, and purposed that these miRNAs might be involved in immune response mechanisms, autophagy and apoptosis (Zheng et al., 2012). These reports shed light on the importance of small noncoding RNA in immunity, but further studies will be required to more fully understand the involvement and mechanism of miRNAs in modulating the host immune response as well as miRNA-based strategies used by *Brucella* for immune evasion.

## Summary and perspectives

Although in brucellosis humans are infected as incidental host, approximately 500,000 new cases reported annually, yet no patient-friendly treatment or effective vaccines available for humans. Additionally, *Brucella* spp. shows deliberate release through direct discharge to poses risk to public health. *Brucella* has remarkable strategies enabling avoidance of the host immune response and facilitating the establishment of chronic infections. However, for survival the host cells have developed complex immune mechanisms to defeat and battle against pathogens and maintain a balance between host resistance and *Brucella* virulence. During the intracellular replication *Brucella* shows typical tissue tropism for lymphoreticular and reproductive systems that helps in evasion of innate and adaptive immune mechanism of host to establish clinical disease manifestations and pathogenesis. At the early stage of infection symptoms observed in humans includes, fatigue, pyrexia, anorexia, myalgia and diaphoresis. However, during chronic stage of brucellosis, persistence occurs in the tissues of mononuclear phagocyte system including bone marrow, lymph nodes, liver and spleen. Likewise it also persists in male reproductive organs, placenta and fetus. At early process of infectious pathogenesis, *Brucella* modulate the immune response mechanism of host to quickly translocate through mucosal immune barrier and is endocytosed by mucosal macrophages and DCs. Stealthy brucellae use different type of strategies to establish and maintain chronic infection by fusion of type IV secretary system dependent BCVs with lysosome to evade intracellular destruction. *Brucella* infects the host cell and protects itself by limiting PRRs including complement system and TLR signaling pathways. *Brucella* LPS shows non-cononical structural differences that inhibit antigen presentation to T cells and dampens innate and adaptive immune mechanisms. Moreover, *Brucella* LPS inhibits activation of DCs to subvert the immune response and establish protective Th1 immune response by secreting IL-12 and preventing T-cell stimulatory action. *Brucella* modulates MHC-I and MHC-II expression prompted by IFN-γ which depend upon cytokine regulation. In addition, *Brucella* uses multiple strategies such as cloaking to avoid recognition by the autophagy mechanism and development of replicative niches. Furthermore, inhibition of apoptosis is another strategy of *Brucella* to evade the immune response to establish chronic infection.

Once *Brucella* adapted to the intra-macrophage environment it extends intracellular replication to reach other systems preferably to target cells and tissues such as skeletal tissues, male genitalia, placental trophoblasts, reticuloendothelial system and endothelium. Newly identified protein UPR and Btp1/TcpB are also important in innate immune evasion during early stages of infection. Recent evidence suggests that Yip1A and IRE1α are essential for *Brucella* replication and rBCV biogenesis that ultimately supports nutrient acquisition and pathogen cell-to-cell spread. Even though numerous strategies employed by *Brucella* to evade the immune response have been identified, there are many questions that need to be answered in respect to other “stealth” mechanisms. What mechanism is involved in apoptosis inhibition by *Brucella* in infected host cells remains to be clarified? How do miRNAs interfere with innate and adaptive immune response mechanisms? How miRNAs cause modulation in *Brucella* intracellular survival? What is the role of miRNAs in autophagy and apoptosis mechanism will be an open question for the next few years. In depth understanding of the “stealth” strategies used by this pathogen will facilitate focus on the pathogenicity of bacterium and development of novel effective therapeutic approaches to treat brucellosis in the future.

## Author contributions

WA and ZL designed the research and wrote the manuscript. KZ helped to prepared the figures. ZL revised the manuscript critically for relevant intellectual content. All authors have read and approved the manuscript.

### Conflict of interest statement

The authors declare that the research was conducted in the absence of any commercial or financial relationships that could be construed as a potential conflict of interest.
